# A Multiscale Material Testing System for In Situ Optical and Electron Microscopes and Its Application

**DOI:** 10.3390/s17081800

**Published:** 2017-08-04

**Authors:** Xuan Ye, Zhiguo Cui, Huajun Fang, Xide Li

**Affiliations:** 1 Department of Engineering Mechanics, Applied Mechanics Lab., Tsinghua University, Beijing 100084, China; ye-x12@mails.tsinghua.edu.cn; 2 Aero Engine Academy of China, Beijing 101304, China; 3 Center for Nano and Micro Mechanics, Tsinghua University, Beijing 100084, China; 4 Suzhou Delve Test Precise-Instrument Co., Ltd., Suzhou 215000, China; ivy001@163.com; 5 Institute of Microelectronics, Tsinghua University, Beijing 100084, China; hjfang@mail.tsinghua.edu.cn

**Keywords:** multiscale, material testing system and force sensor, mechanical property, *Bacillus subtilis* filament

## Abstract

We report a novel material testing system (MTS) that uses hierarchical designs for in-situ mechanical characterization of multiscale materials. This MTS is adaptable for use in optical microscopes (OMs) and scanning electron microscopes (SEMs). The system consists of a microscale material testing module (m-MTM) and a nanoscale material testing module (n-MTM). The MTS can measure mechanical properties of materials with characteristic lengths ranging from millimeters to tens of nanometers, while load capacity can vary from several hundred micronewtons to several nanonewtons. The m-MTM is integrated using piezoelectric motors and piezoelectric stacks/tubes to form coarse and fine testing modules, with specimen length from millimeters to several micrometers, and displacement distances of 12 mm with 0.2 µm resolution for coarse level and 8 µm with 1 nm resolution for fine level. The n-MTM is fabricated using microelectromechanical system technology to form active and passive components and realizes material testing for specimen lengths ranging from several hundred micrometers to tens of nanometers. The system’s capabilities are demonstrated by in-situ OM and SEM testing of the system’s performance and mechanical properties measurements of carbon fibers and metallic microwires. In-situ multiscale deformation tests of *Bacillus subtilis* filaments are also presented.

## 1. Introduction

The development of micro- and nanoscale experimental mechanics has been driven by the need to evaluate the mechanical behavior of materials on small or specific scales. The early driving forces included direct measurement of the performances of microelectromechanical system (MEMS) and micro-scale devices and subsequent analysis and prediction of the reliability of these devices [1,2], along with the development of test methods and instruments to determine how the mechanical properties of materials change when their external dimensions or internal characteristic scales are greatly reduced [3,4,5]. Over the past two decades, when faced with the dramatic volume shrinkage of microelectronic devices [6,7] and the belief in the tenet for materials that smaller is stronger [8,9,10], researchers have made rapid progress in studies of micro- and nanoscale experimental mechanics, including elegant measurement methods with powerful measuring instruments that can be categorized as miniaturized material mechanical testing techniques, MEMS-based and probe-based testing techniques.

In the early 1980s, the rapid development of the semiconductor industry gave new impetus to development of microscale material testing because of the importance of thin films. It was found that the residual stress in a dielectric thin film coated on a wafer surface may even lead to cracking of the film itself. Therefore, it was essential to know the basic mechanical properties of the materials used, such as the Young’s modulus, the fracture strength and the yield stress [11,12]. However, measurement of these mechanical properties was difficult when using traditional experimental methods because of the small dimensions involved. Following macroscopic material mechanical testing techniques, various types of miniaturized material testing machines or devices combined with microscopic and optical methods were developed to evaluate the properties of these films. The earliest techniques included bend testing [13,14,15], bulge testing [16,17,18,19,20], and wafer curvature testing [21]. Subsequently, a large number of important research works enriched this area. Typical examples as Ogawa et al. [22] and Sharpe et al. [23] constructed elegant testing systems to measure the deformation of thin films. Fleck et al. [24] and Stolken et al. [25] performed torsion and bending experiments to confirm the presence of the scaling effect. Zhang et al. [26] proposed a novel microbridge testing method for thin films and provided an analytical deflection versus load solution to evaluate the Young’s modulus, the residual stress and the bending strength of thin films simultaneously. While these miniature material mechanical testing methods are not available for testing of smaller-scale specimens in high-resolution observation systems, such as atomic force microscopes (AFMs), scanning electron microscopes (SEMs), and transmission electron microscopes (TEMs), because they follow macro-mechanical measurement methods, they have been developed further since the early 21st century because they are simple, intuitive, inexpensive and easy to implement [26,27,28,29,30,31].

As the characteristic scale of objects to be tested continues to be reduced even further, the demand for reliable knowledge of material parameters is rising. We need more effective and reliable measurement techniques and platforms for testing of micro- and nanoscale specimens such as ultra-thin films, nanoscale components, nanotubes, nanobars and nanowires. However, the miniature material mechanical testing methods mentioned above are not adequate for accurate measurement of the desired information, despite the fact that they can provide many important basic parameters. Additionally, the main issues, such as preparing the specimens, handling or griping specimens, realizing high-resolution force and displacement measurements, and interpreting the mechanism of the interaction or coupling between the small specimens and the test system or test environment, in micro- and nanoscale testing make the experiments extremely difficult. To realize micro- or nanoscale tests and obtain accurate material parameters, researchers start to develop their own test devices and methods, which can be categorized into MEMS-based and probe-based testing techniques.

As an important technology in the 20th century, MEMS can be used to produce complex structures, devices and systems on a micrometer scale [32]. Small-scale MEMS technology thus offered the opportunity to characterize micro- to nanoscale materials that would not normally be available for miniature material testing devices. A number of developed MEMS-based instruments were thus dedicated to micro- and nanomechanical experiments. Typical works included those of Zhu and Espinosa, in which they systematically developed integrated test systems using MEMS for in situ electron microscopy mechanical testing of nanostructures [33,34,35,36]. Haque and Saif introduced MEMS platforms to characterize nanoscale thin films inside a SEM and a TEM [37,38,39,40,41]. In addition, more extensive research based on the use of MEMS technology and platforms to study the mechanical properties of films and one-dimensional materials has been reported [11,42,43,44,45,46,47,48,49,50,51,52]. MEMS-based testing techniques are powerful tools for mechanical characterization of micro- and nanoscale materials that integrate an actuator to deliver a load or displacement to the sample and a sensing unit to measure the load on a chip. The advantages of this technique include ease of clamping and loading and high-accuracy deformation detection, while the disadvantages include expensive and complex manufacturing processes, and sample size and material type limitations.

Probe-based testing techniques combine various probe units and experimental systems to implement mechanical testing of materials and structures at the micro- or nanoscale [53]. The probe can be a microcantilever, such as that used in AFMs [54], a straight bar (i.e., a chemically etched tungsten wire or a hot-drawn glass rod) that has a sharp tip combined with a manipulator [55], and an indenter such as that used by a nanoindenter [56,57]. The probe can not only be used as a stick to load and manipulate objects, but also has additional features such as the ability to sense force [58], temperature, and other physical parameters [59]. The probe is always combined with a micro-/nanomanipulator, or is attached to a translation stage during the tests. There are various modes for probe-based mechanical tests. One of these modes is similar to an indentation test and consists of three phases: the probe tip approaching the tested surface, indenting and contacting the surface (i.e., loading), and retracting from the surface (i.e., unloading) [60]. These three phases can be used to investigate the probe tip-surface system interaction, the local mechanical properties of the tested material, and the mechanical behaviors of the probe and the tested structures, respectively [61,62]. Other modes include manipulation of the probe to implement mechanical testing of micro- and nanoscale materials and components, and investigation of the interactions between the probe and the surfaces/interfaces of thin films or biological materials when using the stages of the optical microscopes (OM), AFM, SEM, and TEM. Examples include the thermally- or electrostatically- induced vibrations inside TEMs [63,64], lateral bending using AFM probes [65], in situ tensile testing inside an SEM [66], and manipulation of micro graphite flakes under both an OM and an SEM [67,68,69]. Probe-based techniques are relatively new to experimental mechanics. Some methods and systems have been developed to facilitate measurement and manipulation of micro-/nanoscale objects, and probe-based testing apparatuses have been made on stages of OM, AFM, SEM and TEM. These devices are indispensable for micro-/nanomechanical testing because of their ease of operation and their extraordinarily high load and displacement resolutions. However, some intractable problems still remain in this area, including clamping, loading, manipulation, and microforce and displacement sensing.

MEMS-based and probe-based techniques increasingly tend to be applied to smaller and smaller objects, and this is accompanied by dramatic shrinkage in the volumes of materials and the novel mechanical properties that are demonstrated at such greatly reduced scales. The advantages of these approaches are that they can provide delicate equipment and achieve fine force and displacement measurements for samples with specific dimension requirements. However, because they are designed for limited measurement ranges and simple functionality, these devices are not suitable for measurement of multiscale materials, such as biological materials, composite materials, and devices with complex structures, with dimensions that can range from more than millimeters down to the micrometer and nanometer scales. In this study, we design a novel material testing system (MTS) that uses hierarchical designs for the in situ mechanical characterization of multiscale materials in OMs and SEMs. The MTS consists of a microscale material testing module (m-MTM) and a nanoscale material testing module (n-MTM), and can measure the mechanical properties of various materials with characteristic lengths ranging from millimeters to tens of nanometers, while the load capacity can vary from several hundred micronewtons to several nanonewtons. The system capabilities are demonstrated by in situ OM or SEM testing of the system’s performance and by performing mechanical properties measurements of carbon fibers and metallic microwires. The specialty of the system is embodied in the tensile testing of multiscale *Bacillus subtilis* (*B. subtilis*) filaments/threads. We also expect this hierarchical design to be useful in the study of the multiscale mechanical properties of other materials, and that the multifunctional features that combine the capabilities of miniature material testing machines and probe-based and MEMS-based testing devices will be suitable for manipulation, loading, and clamping of micro- and nanoscale materials in other areas.

## 2. Instrumentation

As mentioned above, the three available types of measuring devices provide an abundance of platforms to perform mechanical measurements at the micro- and nanoscales. However, as specific measurement devices, in addition to their limited measurement ranges and simple functions, another important limitation of the individual measuring devices is that additional auxiliary systems must be provided during the measurement process for movement, positioning, and manipulation of the samples. This increases the difficulty of the test process, reduces the reliability of the devices, and is not conducive to either promotion or synergistic use of these systems. Our design strategies are: (1) use of a platform-based multi-level modular design to measure material mechanical properties at both the mm-to-μm scale (with the m-MTM) and the μm-to-nm scale (with the n-MTM); (2) use of piezoelectric stacks and piezoelectric ceramic tubes to achieve sample loading and manipulation in both Cartesian and polar coordinates, respectively; (3) integration of both coarse and fine multi-modules, along with separate control of the displacement or movement at the micrometer and nanometer levels to ensure that the developed MTS has the functions of each of the small-scale MTS, MEMS-based, and probe-based instruments. In this section, the specific design and capabilities of the developed MTS are introduced. The entire test system consists of two main modules: the m-MTM and the n-MTM. The former is used to measure the mechanical properties of various materials with characteristic lengths ranging from millimeters to several tens of micrometers, while the latter is designed for materials with characteristic lengths ranging from several hundred micrometers to tens of nanometers.

### 2.1. Microscale Material Testing Module

The m-MTM is designed to perform in situ mechanical tests including tension, compression, and bending tests in both an OM and an SEM (Quanta 450 FEG, FEI, Hillsboro, OR, USA). Experiments are conducted in the OM when the specimen dimensions are large enough, while they are performed in the SEM when the specimens are so small that the diffraction-limited spatial resolution of the OM is insufficient. The m-MTM is connected to a control and data acquisition system outside the SEM using a specially designed flange with a feed-through. One-dimensional or two-dimensional materials on the micro- or nanoscales can be measured when the m-MTM is combined with the n-MTM.

**Figure 1:**
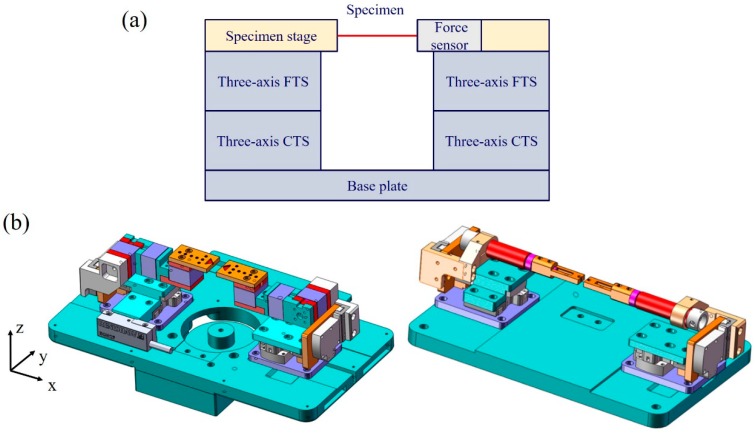
(**a**) Schematic diagram and (**b**) computer-aided design (CAD) of the developed microscale material testing module (m-MTM). Two sets of *xyz*-axis coarse translation stages (CTSs) and two sets of three-axis fine translation stages (FTSs) (left: piezoelectric stacks; right: piezoelectric ceramic tubes) are present on the base plate.

**Figure 2:**
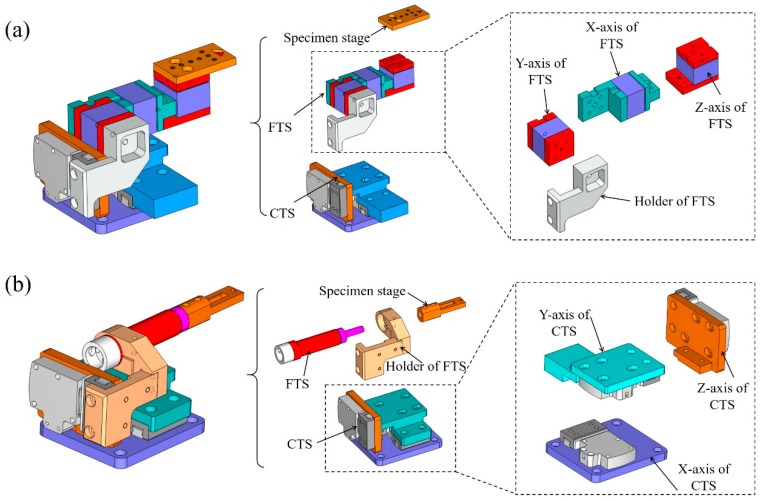
CAD structure diagrams of the m-MTM highlighting the assembly diagrams for the two-level structures. (**a**) Piezoelectric stacks; (**b**) Piezoelectric ceramic tubes.

A schematic diagram and a computer-aided design (CAD) of the developed m-MTM are shown in Figure 1a,b. The main components of the m-MTM are two sets of three-axis (*xyz*-axis) coarse translation stages (CTSs) and two sets of three-axis fine translation stages (FTSs) that allow the system to realize displacement loads ranging from millimeters to nanometers. The *xyz*-axis CTSs and the corresponding translational piezoelectric guides are installed on a support unit (i.e., the base plate), and the three-axis FTSs are installed at the upper end of the *z*-axis of the CTSs. The *xyz*-axis CTSs use piezoelectric motors with a working distance of 12 mm and displacement resolution of 0.2 μm, and the FTSs use piezoelectric stacks or piezoelectric ceramic tubes with a working distance of 8 μm and displacement resolution of around 1 nm. The separate use of the piezoelectric stacks and the piezoelectric ceramic tubes in the FTSs enables movement and loading with Cartesian coordinates (*x*, *y*, *z*) or movement, loading, and manipulation with polar coordinates (*α*, *φ*, z), respectively. Figure 2a,b show the CAD structure diagrams and the corresponding assembly diagrams, respectively. The m-MTM works via a displacement-control mode and the load cell can be connected to one of the free ends (*x*-axis) of the FTSs.

**Figure 3:**
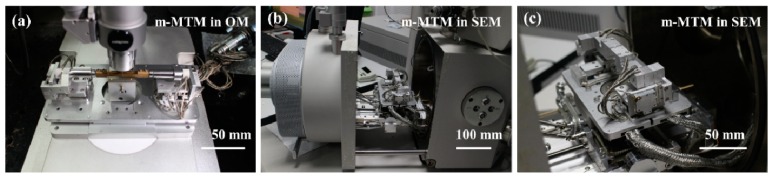
Photographs of the m-MTM used with (**a**) an optical microscope (OM) and (**b**,**c**) a scanning electron microscope (SEM).

Given the diversity of the materials under test and the specimen sizes, the displacement loading rate can be regulated over a range from 10 μm/s to 10 nm/s. Figure 3 shows photographs of the m-MTM during use with the OM and the SEM. To fulfill all the constraints of the SEM and minimize the pump down time, only vacuum-compatible materials with low magnetic susceptibility that prevent interference with the electron beam are used. The m-MTM is supported on a standard aluminum alloy plate with dimensions of 175 × 95 × 7 mm^3^ that can be connected to the SEM sample stage using two hex screws (in the vacuum chamber) or can be placed independently on the object stage of the OM. The dimensions (length × width × height) of the piezoelectric stack are 10 × 10 × 10 mm^3^. The piezoelectric tube has a length of 30 mm, an outer diameter of 6.35 mm and an inner diameter of 5.35 mm. These piezoelectric devices are selected according to the working distance, the control voltage and the control accuracy.

To realize the multiscale force measurement and meet various functional requirements, we design several types of exchangeable force sensors. For the relatively large load measurement, a strain gauge force sensor is made with a force range of 200 mN and a resolution of 0.1 mN. The linearity and repeatability is 0.1% of the full scale. Also, the average stiffness coefficient is 1.25 mN/μm. To perform microforce sensing and loading during the micromanipulation, we developed a sequence of single-cantilever piezoresistive force sensors to cover a broad range of forces from 200 mN to 37.5 nN [58].The m-MTM is also suitable for some commercial force sensors, such as a probe-type capacitive microforce sensor (FT-S100, FemtoTools, Buchs, Switzerland) with a force range of 100 μN and a resolution of 5 nN. Above micro force sensors are calibrated before tests by electronic speckle pattern interferometry [58] and magnetic levitation vibrator calibration system [68,70]. Specifically, the m-MTM and the strain gauge force sensor combined to characterize the performance of the multi-scale integrated system, including tensile tests of carbon fibers and metallic microwires. The m-MTM was also combined with the single-cantilever force sensor to achieve the multi-scale mechanical properties of *B*. *subtilis* fibers.

### 2.2. Nanoscale Material Testing Module

Measurement of the mechanical properties of materials and structures with characteristic lengths ranging from hundreds of micrometers to tens of nanometers requires a specially designed n-MTM, which can be a complex and extremely expensive module. Therefore, we propose a modular design strategy with separate control that allows commercial or self-designed n-MTMs to be used in the developed MTS. For example, the passive push-to-pull (PTP) module (Hysitron, Inc., Minneapolis, MN, USA), which is fabricated on a silicon-on-insulator wafer with four identical supporting springs and a central gap for specimen placement [71,72], can be combined with our m-MTM to stretch or compress the specimens. In this case, the left specimen stage (LSS) of the m-MTM is used to provide the support, movement and positioning of the PTP module, while the right specimen stage (RSS) can be used to push the semicircular end of the PTP module to realize uniaxial tension or compression testing of materials from micro- to nanoscales. Similar arrangements can also be used for active n-MTMs such as the on-chip systems developed by Zhu and Espinosa [34], Haque and Saif [38], or Hazra and Baker [45]. These active devices include an actuator (either electromechanical or thermomechanical) and a load sensor (based on capacitance, the piezoresistive effect or the resistance change caused by deformation) and can complete the tests independently without need for external loading or force sensing. In this manner, the developed m-MTM can be used exclusively in supporting, fixing, orientation, and manipulation of these active n-MTMs, such that one of the m-MTM’s specimen stages supports, fixes, or orientates the active n-MTM, while the other stage is connected to a probe (i.e., the FTS using a piezoelectric tube) to provide the necessary manipulation and clamping of the specimen under test.

**Figure 4:**
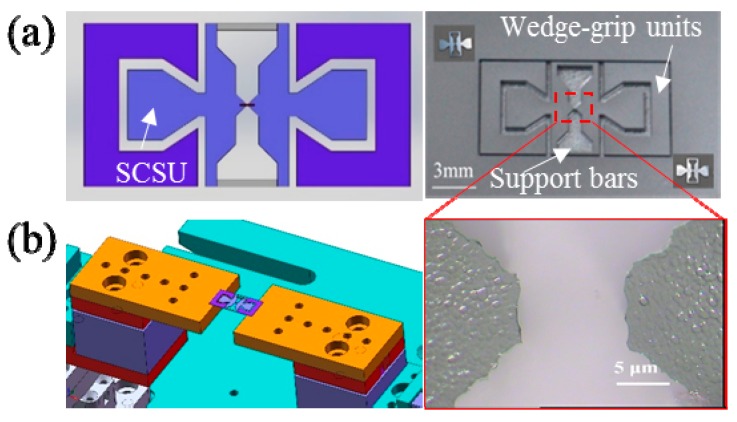
(**a**) Design and photograph of a passive nanoscale material testing module (n-MTM) for tensile testing; (**b**) Passive n-MTM fixed on the specimen stages of the m-MTM. The enlarged photograph shows the 10 μm specimen space of the specimen connection and support unit (SCSU).

Here, we design several types of n-MTM that can be divided into passive and active on-chip modules. Figure 4a shows a simple passive module for use in tensile tests. The module consists of a pair of symmetrical wedge-grip units and a specimen connection and support unit (SCSU). This passive module is manufactured on a silicon wafer using well-known silicon deep etching technology [73]; the process includes procedures for deposition of an oxide layer on the wafer surface, gluing and exposure on the front side, development, deep reactive ion etching (DRIE), removal of the photoresist, and release of the structure. In practical tests, we first fix the symmetrical wedge-grip units to the specimen stage of the m-MTM, and the specimen is then installed on the specimen end of the SCSU. Usually, the specimen clamping method can be electron beam-induced deposition (EBID), van der Waals interaction, capillary adhesion, or gluing, depending on the specimen under test and the actual measurement environment. Finally, the SCSU is inserted into the wedge-grip units to prepare for testing. Sometimes, to make the experimental procedure easier, the SCSU can be installed on the wedge-grip units first before the specimen is clamped to the specimen end of the SCSU, e.g., for experiments in the SEM. Afterwards, the double support bars of the SCSU are cut off using a tiny rotating diamond saw, and the m-MTM then provides external force loading by uniaxial stretching or compression of the passive module to complete the measurements. There are two specimen space sizes of 10 μm and 100 μm in the SCSU. The load is recorded using a load cell and the corresponding displacement can be obtained through analysis of the OM or SEM images during the tests. Figure 4b shows a passive n-MTM fixed on the m-MTM specimen stage. The enlarged photograph shows the 10 μm specimen space in the SCSU.

**Figure 5:**
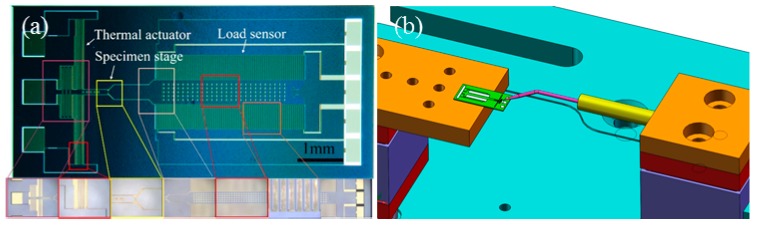
(**a**) Active n-MTM, including actuator, load sensor, and specimen stage; (**b**) An active n-MTM is fixed on one specimen stage of the m-MTM, while the other specimen stage is used to manipulate and grip the specimen.

To measure even tinier specimens, a more sophisticated active n-MTM is also fabricated recently using silicon deep etching technology. This unit integrates a thermal actuator, a load sensor, and a specimen stage for placement and support of the tested specimen (Figure 5a). Two specimen space sizes of 2 μm and 10 μm are designed. Tensile loading is achieved by Joule heating of the polycrystalline silicon chevron beam to produce uniaxial thermal expansion displacement. The force values during testing are sensed using a comb-like capacitive force sensor. During practical experiments, the n-MTM chip is placed on one specimen stage of the m-MTM, which then provides support and positioning for the n-MTM chip. The other m-MTM specimen stage can then be used to manipulate and grip the specimen, or to perform auxiliary operations during the experiments (Figure 5b). The n-MTM is connected to a power supply, a signal acquisition circuit, and a computer located outside the SEM via a chamber feed-through. Detailed performance data and applications of this n-MTM will be described in another paper.

Force sensors with different stiffness are fabricated by varying the designs of the folded beams. When the sensor is supported by four pairs of double-folded beams, the sensor’s stiffness is calculated to be $K_{S}=24EI/l^{3}=2{Eb}^{3}h/l^{3}$, and when it is supported by four pairs of triple-folded beams, the sensor stiffness is calculated to be $K_{S}=16EI/l^{3}=4{Eb}^{3}h/3l^{3}$; here, *l* is the folded beam length, *b* is the folded beam width, and *h* is the folded beam height. The typical stiffness value of the double-folded beam is 37.3 N/m, with *l* = 500 μm, *b* = 7 μm, and *h* = 40 μm. Because the integrated sensor displacement resolution is 2 nm and its range is 4 μm (following the procedure given in [36]), the sensor force resolution is 74.6 nN and its range is 149.2 μN. For a triple-folded beam with the same dimensions, the sensor stiffness is 24.9 N/m, the force resolution is 49.8 nN, and the force range is 99.6 μN.

### 2.3. Control System and Feedback Operation

To realize different testing modes (biaxial tension or compression), bidirectional symmetric modules and corresponding control units (three-dimensional movement control, position detection, and feedback system) are utilized in the m-MTM. A control and data acquisition system based on digital signal processor (DSP) is used and the control interface is connected to computer through a USB 2.0 link for expediently receiving commands from the operator.

Before any desired test is conducted, the corresponding control mode should be chosen to operate the transducer. The control unit is used to set the initial value of digital-to-analog converters (DACs, analog input/output). Then the linear motor actuators carry out actuating of piezoelectric linear motors. The piezoelectric linear motors provide the coarse displacement of the m-MTM (coarse control), and piezoelectric actuators provide fine displacement of the m-MTM (fine control), separately. For coarse control, grating system is used to acquire changes of displacement and speed which are fed back to DSP, while for fine control, the data acquisition of piezoelectric actuator is from the resistance strain gauge.

## 3. Characterization of the System Performance

### 3.1. Stability and Drift of the System

The stability and performance of any micro-/nanoscale experimental system must be ensured and demonstrated before testing, because the detected force and displacement must be of the same order or below the level of the perturbations from the test environment (e.g., air disturbances, thermal emissions, vibrations, and electromagnetic interference). Because this system needs to work in a high vacuum environment and perform mechanical measurements with sample characteristic lengths ranging from a few millimeters to tens of nanometers, it is challenging to ensure the stability of the mechanical structure and also solve the electromagnetic interference problem. In this section, the results of several experiments performed to characterize the drift of the system and its influence on the measurement accuracy are presented.

#### 3.1.1. Stability and Drift of m-MTM

To meet multi-scale measurement requirements, the system must have excellent resistance to drift under various loading conditions. The instrumental drift is defined as “continuous or incremental change over time in indication because of the changes in metrological properties of a measuring instrument” [74]. After the m-MTM was tightly installed on the SEM sample stage, stable and unambiguous images could still be obtained at 80,000× magnification. Subsequently, we examined the system drift under a variety of loading conditions (i.e., no load, moderate load, and high load) and evaluated its effects on the measurement accuracy. The homemade strain gauge force sensor was used and connected to the RSS using two hex screws. Because the SEM sample stage and the primary electron beam always move slightly within the SEM chamber [75,76], the amplitude of the spontaneous drift must be measured.

**Figure 6:**
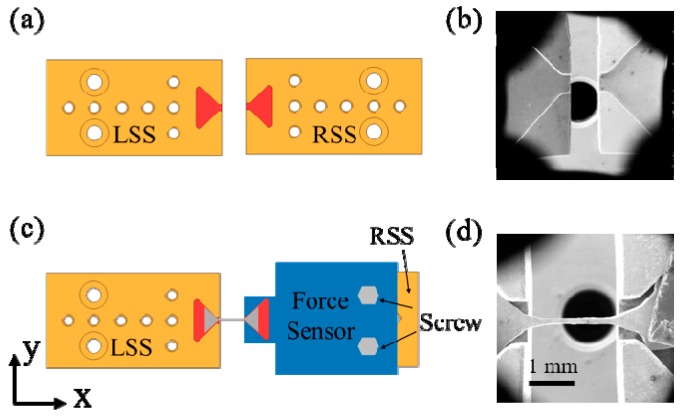
(**a**,**b**) Schematic diagram and photograph of the m-MTM specimen stages under no load conditions; (**c**,**d**) Schematic diagram and photograph of the left specimen stage and the force sensor when connected using a dog bone-shaped specimen.

**Figure 7:**
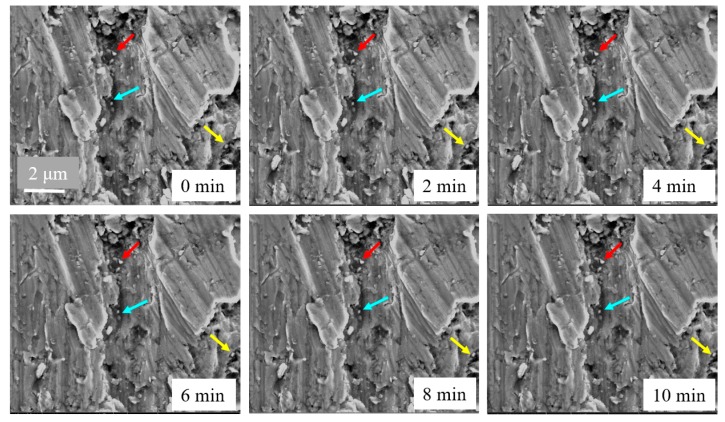
Typical SEM image series of the left specimen stage (LSS) of the m-MTM under 11 mN loading at 10,000× magnification. The natural markers on the LSS are selected as the tracked points (pointed by the arrows).

The drift of the m-MTM applied force with initial values of 0, 0.5 mN, and 11 mN was measured to ensure suitability for multiscale measurement demands. An almost rigid dog bone-shaped specimen was designed such that both of its ends were stuck in the slots on the specimen stage of m-MTM and force sensor (Figure 6). By controlling the deformation of the specimen, varying loads can be applied and recorded using the force sensor. After the same region on the LSS is scanned (Figure 7) every 1 min at 10,000× magnification, and the natural markers are selected as the tracked points (pointed by the arrows in Figure 7). The motion pattern is tracked through the image series over 15 min (a typical tensile test duration is usually less than 15 min), then the relationship between the systematic drift and the time can be determined. The drift speeds are obtained by linear fitting within 15 min. In the SEM, the detection of the system drift is carried out after the vacuum procedure is completed and the force sensor reading is stable. The drift speed of the image is the drift speed of the system, including the applied force sensor.

The drift speeds under the different loading conditions are summarized in Table 1. Also, we repeated the tests after 30 min under four different loading conditions. Corresponding drift speeds are listed in Table 2. We found that the drift speed decreased obviously, that the system was stable after 30 min, and that the average drift speed along the loading direction was 4.8–15.9 nm/min (at 10,000× magnification).

In the OM, the m-MTM is fixed on an object stage to minimize the effects of external vibrations. The system drift measurements are then repeated under the same four loading conditions that are used in the SEM. The results show that the drift within 15 min is less than 1 pixel (resolution with a digital optical microscope, where 1 pixel corresponds to a different actual space size at different magnifications) and demonstrate that the system is sufficiently stable.

#### 3.1.2. Stability of the Force Sensors

**Figure 8:**
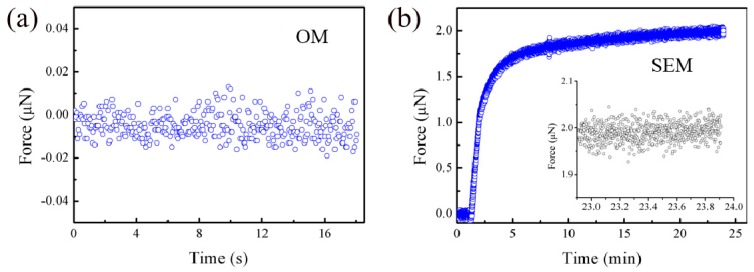

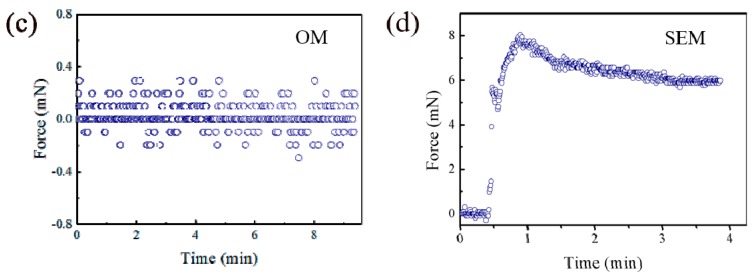
Time-dependent force values of the stationary sensors at room temperature. Zero drift of the FT-S100 force sensor under (**a**) the OM and (**b**) the SEM. The measurement starts at ambient pressure and is maintained during the pumping of the vacuum chamber until the reading is stable. Zero drift of the strain gauge force sensor under (**c**) the OM and (**d**) the SEM.

To ensure that the force sensors are stable in both the OM and the SEM, the stabilities of two typical sensors (a commercial FT-S100 force sensor and a homemade strain gauge force sensor) were characterized at room temperature. The stability of a measurement instrument is defined as a “property of a measuring instrument whereby its metrological properties remain constant in time”. When used in the OM, the m-MTM, which is equipped with bare sensors, is fixed on an optical table and covered using an airtight container to eliminate noise as completely as possible. The time-dependent force values of the stationary sensors are then recorded to represent the stability (Figure 8a,c). When used in the SEM, the entire system is installed on the SEM sample stage and the force sensors are shielded using aluminum enclosures to prevent any interference from the electron beam. During evacuation of the vacuum chamber, readings of the load-free sensor are taken until they become stable (Figure 8b,d).

The results show that evacuation of the vacuum chamber in the SEM can cause serious load drift; however, after evacuation is completed, the sensor enters a stable range after a few minutes. The noise levels for the strain gauge force sensor (calculated as the standard deviation) are approximately 88 μN in air and 112 μN under a high vacuum. For the FT-S100 force sensor, the noise level in air is approximately 6 nN, which is close to the noise level of the sensor datasheet 5 nN. In the vacuum, this level increases to 20 nN.

The noise levels in the SEM are higher than those in the OM, mainly because of electromagnetic interference. The dramatic changes in the zero points during pumping (Figure 8b,d) are attributed to internal components inside the sensors being affected by rapid pressure changes and component deformation.

### 3.2. Demonstration of System Repeatability

In this section, the repeatability of the system is demonstrated using the in situ tensions of two reference materials: carbon fibers and pure copper wires. The repeatability of the measurement results is defined as the “closeness of the agreement between the results of successive measurements of the same measurement carried out under the same conditions of measurement”. A reference material is defined as a “material or substance, one or more of whose property values are sufficiently homogeneous and well established to be used for the calibration of an apparatus, the assessment of a measurement method, or for assigning values to materials”.

#### 3.2.1. Tension of Individual Carbon Fibers

**Figure 9:**
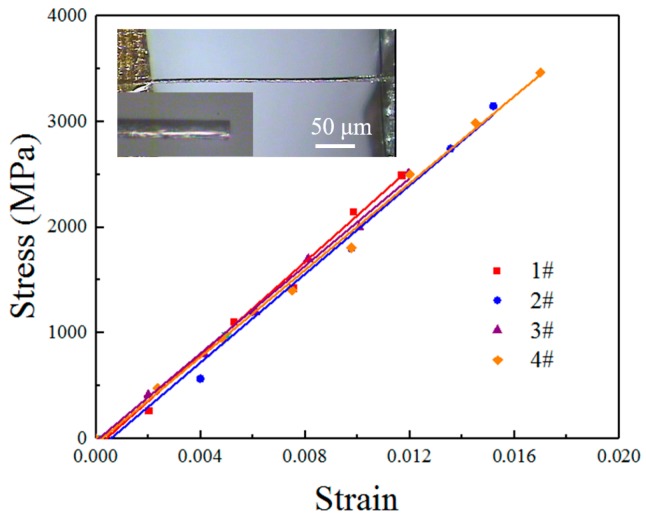
Tensile results for T300 fibers. The insets show the typical fracture morphology under the OM.

Carbon fiber manufacturing technology is quite mature, and thus carbon fiber’s performance is stable and its mechanical parameters are clear and are well known. We selected 7-μm-diameter T300 (T300B-6000-50B, Toray, Tokyo, Japan) fibers to be used as a reference material. In the OM, a complete experimental system is established based on the m-MTM that uses the strain gauge force sensor as the force detector, and it uses the mark point method and image sequences to detect the distance between the two mark points (used to measure the displacement of the specimen). Alignment of the specimens can be achieved easily by three-dimensional manipulation of the m-MTM. Four groups of tensile results (with an average length of 500 μm) are listed in Table 3, and the tensile curves indicate that the T300 fiber is a type of elastic brittle material (see Figure 9). The measured properties of these fibers are tensile modulus of 208 ± 16 GPa, tensile strength of 3.1 ± 0.5 GPa, and elongation of 1.4 ± 0.2%, which agree well with previously given values (http://www.torayca.com/en/index.html). The repeatability of these results demonstrates the good performance of the m-MTM. Additionally, the flat fracture morphology (inset of Figure 9) shows that the sudden fracture is derived from the defects and initial cracks in the fibers. When the load exceeds a specific threshold, brittle cracks propagate rapidly. The tensile strength is restricted by the surface defects (e.g., by grooves on the surface) and by internal voids.

#### 3.2.2. Tension of the Individual Copper Wires

**Figure 10:**
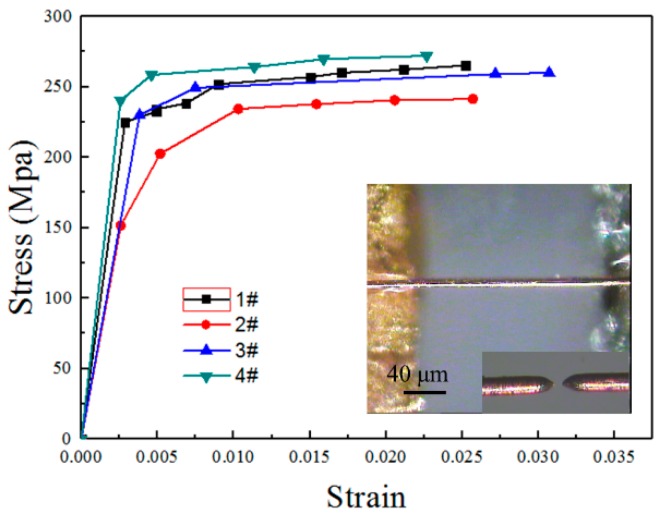
Tensile results for copper wires. The inset shows the typical fracture morphology, which indicates that it is an ideal plastic and pure shear fracture.

Basing on the same experimental system in Section 3.2.1, four 20 μm-diameter copper wires (99.99% purity, annealed; Goodfellow Cambridge Ltd., Huntingdon, UK) are stretched and show typical elastic plastic properties and fracture after local necking (Figure 10). The measured elastic modulus is 72.6 ± 12.7 GPa, the tensile strength is 259.8 ± 10.2 MPa, the yield strength is 224.5 ± 12.3 MPa, and the fracture strain is 2.6 ± 0.3% (Table 4), which are close to the reference values for hard copper (which are provided by the manufacturer). The tensile strength is higher than that of the macroscopic copper (where the tensile strength is 50–70 MPa), and the fracture strain is lower than that of macroscopic copper (where the fracture strain is 40–50%), which is mainly related to the small sample size. The good repeatability of the results demonstrates that the m-MTM is reliable.

**Figure 11:**
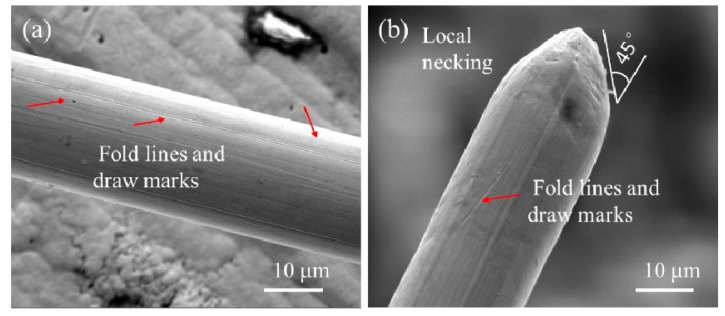
Typical surface and fracture morphology of copper wires under the SEM.

At the micron scale, the plastic deformation is closely related to the sample microstructure, e.g., its grain size and crystal orientation. From the stress-strain curves, the degree of work hardening at this stage is lower than that achieved at low annealing temperatures (e.g., 200 °C-annealed sample) [77]. We may speculate that the grain size is small based on the Hall-Petch effect. The surface oxidation layer may contribute to the high yield strength of the sample by blocking internal dislocation nucleation within the wire. While the SEM image (Figure 11) shows that the diameter is uniform and that the surface quality along the axis of the wire is good, obvious fold lines and draw marks from the manufacturing process exist on the surface, and this may lead to premature local necking in the middle of the specimen and small fracture strain.

## 4. Applications

At present, the fabrication of new biological fibers as substitutes for the natural or chemically synthesized fibers that are used in daily or engineering applications is pursued by biologists and materials researchers. The filamentous bacterium *B. subtilis* has emerged with the large possibility for use as a cotton-like material. The diameter of an individual *B. subtilis* filament (which contains hundreds of cells that are connected by septa) is only a few hundred nanometers, while the diameters of natural fibers such as silk are approximately one order of magnitude larger (5–7 μm) [78]. For practical applications, *B. subtilis* filaments should be weaved together to form bacterial threads (that contain hundreds of parallel filaments) with millimeter-scale diameters. Therefore, as promising engineering materials, it is necessary to understand their deformation behavior and some of their basic mechanical properties over the range from individual bacterial filaments to bacterial threads.

Some of previous measurement methods focused on the single cell scale. For example, combining the force–penetration curves obtained via AFM with nanoindentation theory, elasticity parameters of the bacterial cell were obtained [79,80]. AFM provides information with very high spatial resolution, but only measures the properties of the cell wall. Sun et al. [81,82] realized the mechanical property characterization of a single cell based on the MEMS technology by point loading. Further, a set of MEMS devices for cell mechanics have been reviewed [83]. MEMS-based methods allow accurate transduction of forces and displacements, but are only suitable for cellular and subcellular level mechanical testing. And some methods focused on the scale of bacterial threads. For example, Thwaites et al. [84] stretched bacterial threads containing 20,000 parallel filaments by textile testing. Their methods are not suitable for predicting the mechanical properties of the cell wall and individual cellular filaments. None of above testing results revealed the failure mechanism of this multilevel and multiscale material. Here, we use the developed MTS to study multiscale mechanical properties of *B. subtilis* over the range from individual bacterial filaments (which contain several to several hundred bacterial cells) to bacterial threads (which contain hundreds of filaments in a parallel alignment) under tensile loading. We stretch the individual bacterial filaments and bacterial threads directly using a novel manipulation procedure called the liquid drop method (LDM) on the m-MTM combining with the single-cantilever force sensor on an OM platform, and investigate deformation behavior of short bacterial filaments consisting of several cells on the n-MTM in an SEM chamber.

### 4.1. Deformation Tests of Individual B. subtilis Filaments

The results of a tensile study of individual *B. subtilis* filaments with an average diameter of 0.7 µm and a length range of 48.9–180.7 µm (from several to several hundred bacterial cells) have previously been presented in detail [85]. The test results indicated that it was a moisture-sensitive type of material that exhibited different mechanical behavior under wet and dry conditions. The filaments showed elastic-brittle behavior under dry conditions, and the average fracture stress and elastic modulus of filaments with the *sigD*, *lytE*, and *lytD* genes deleted were 62.2 MPa and 5.8 GPa, respectively. However, the filaments showed viscoelastic behavior under wet conditions, with an average elastic modulus of 64 ± 15 MPa and an average viscosity of 110 ± 60 MPa s.

**Figure 12:**
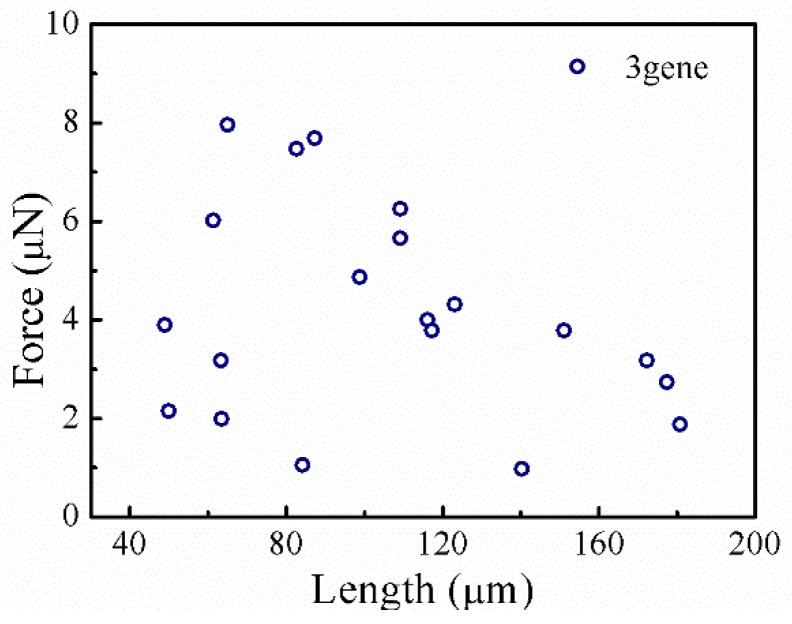
Relationship between fracture load and sample length of *B. subtilis* filament with *sigD*, *lytE* and *lytD* genes deleted.

The test results show that the fracture load decreases rapidly with increasing filament length (Figure 12) and that these filaments have highly variable mechanical properties as a result of weak interactions among the pairs of microbial cells that are known as septa. Higher numbers of septa in a filament will cause their mechanical properties to vary to a greater extent. In individual filaments, fractures occur at the positions of septa at low relative humidity (RH), while necking instability comes from the septa at high RH. Additionally, the mechanical properties of the filaments are found to be highly dependent on the RH, with a brittle-ductile transition occurring at around RH = 45%. The mechanism for this brittle-ductile transition is related to the presence of many hydrogen bonds between the peptides of peptidoglycan contained in the filament, which make the polymer networks rigid under dry conditions. As the degree of hydration increases, water then competes for the hydrogen bond sites, which causes the networks to become more flexible.

### 4.2. Deformation Tests of Short B. subtilis Filaments

**Figure 13:**
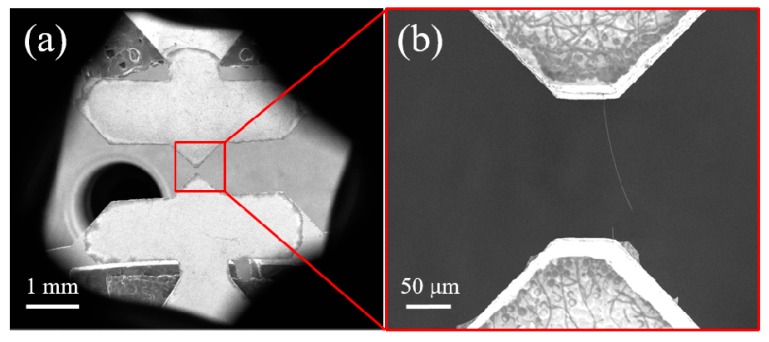
Tensile testing of short *B. subtilis* filaments in SEM. (**a**) In SEM, the passive n-MTM fixed on the specimen stages of the m-MTM; (**b**) A filament is clamped on the specimen ends of the SCSU and then pulled.

Due to the spatial resolution of the OM, the deformation evolution of the filaments cannot be clearly observed, so we stretched short bacterial filaments consisting of several cells on the passive n-MTM in an SEM chamber. After transferring the filaments to the passive n-MTM and waiting for the droplets to evaporate, the filaments are clamped on the specimen end of the SCSU. Then the double support bars of the SCSU are cut off using a tiny rotating diamond saw. We stretch the filaments by using the fine modules of the m-MTM, and observe the deformation and fracture of filaments in real time (Figure 13).

**Figure 14:**
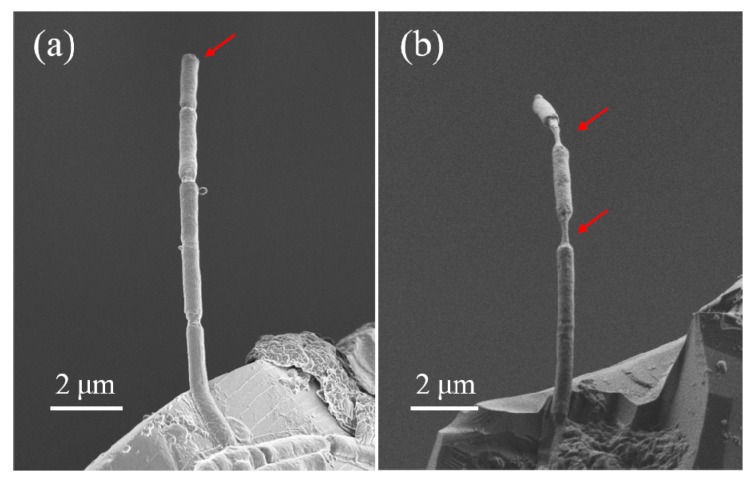
Tensile results of short *B. subtilis* filaments at low RH (**a**) and high RH (**b**) in SEM.

Typical tensile results are shown in Figure 14. At low RH, the fracture occurs only at the position of the septa (Figure 14a). At high RH, the filament shows significant elongation both in the part of the cell wall and at the positions of septa, and ultimately it necks at the positions of septa (Figure 14b), which is consistent with the calculated results of finite element analysis [85].

### 4.3. Deformation Tests of Individual B. subtilis Threads

**Figure 15:**
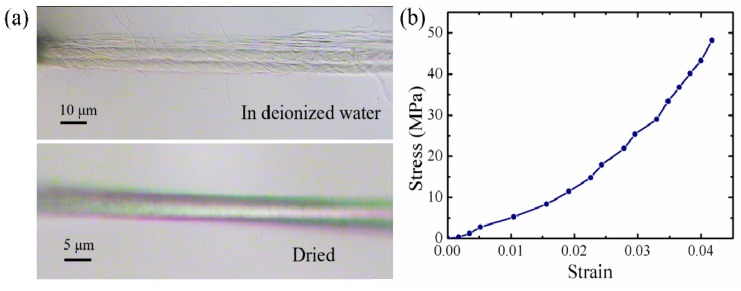
Tensile testing of a typical *B. subtilis* thread. (**a**) Initially, the *B. subtilis* thread suspends in deionized water, before the tensile test, it is dried by the liquid drop method; (**b**) Stress-strain curve of the *B. subtilis* thread.

A typical thread is compressed into a compact and aligned structure by surface tension (Figure 15a), with a length of 564.7 μm and an average diameter of 4.5 μm. The stress-strain curve indicates a nonlinear relationship and brittle fracture (Figure 15b). The fracture strength and strain are 50.0 MPa and 4.0%, respectively. There is no obvious improvement in the fracture strength when compared with individual *B. subtilis* filaments, suggesting that adhesion and friction between the filaments do not contribute significantly to the fracture strength of the thread. Presumably, the individual filaments that are contained in the thread do not achieve a closely-packed alignment structure and shrinkage compaction during the drying process. Therefore, the thread still exhibits mechanical behavior similar to that of individual filaments. The relatively large nonlinear deformation indicates that the internal structure of the thread may not be completely dry.

**Figure 16:**
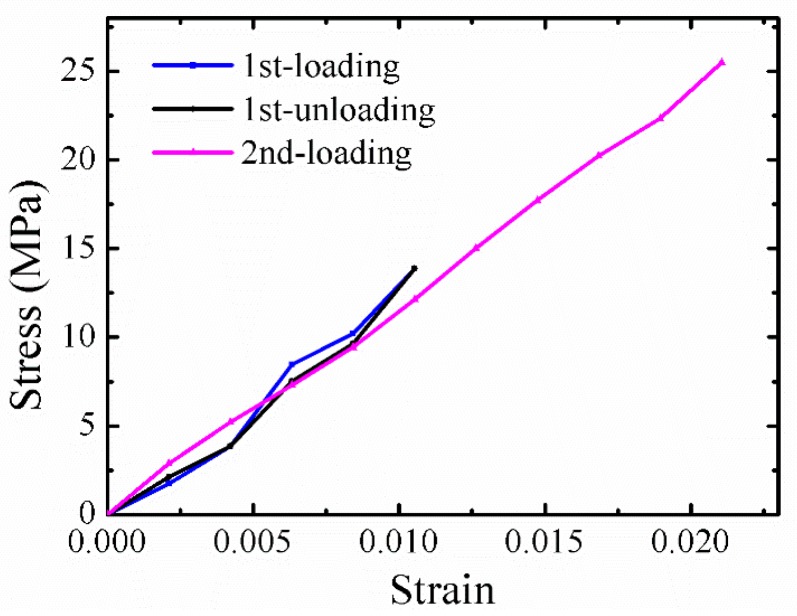
Loading-unloading curves for a typical *B. subtilis* thread.

For a better understanding of the mechanical behavior of *B. subtilis* thread, loading and unloading experiments were also performed. Figure 16 shows typical loading and unloading curves for a *B. subtilis* thread with a length of 466.5 μm and an average diameter of 5.7 μm. After the loading-unloading process, the thread is then stretched to fracture. The loading-unloading curves indicate that it is linear elasticity. Obviously, this is a completely dry thread. The fracture strength and strain are 25.5 MPa and 2.1%, respectively, and are thus not significantly improved.

**Figure 17:**
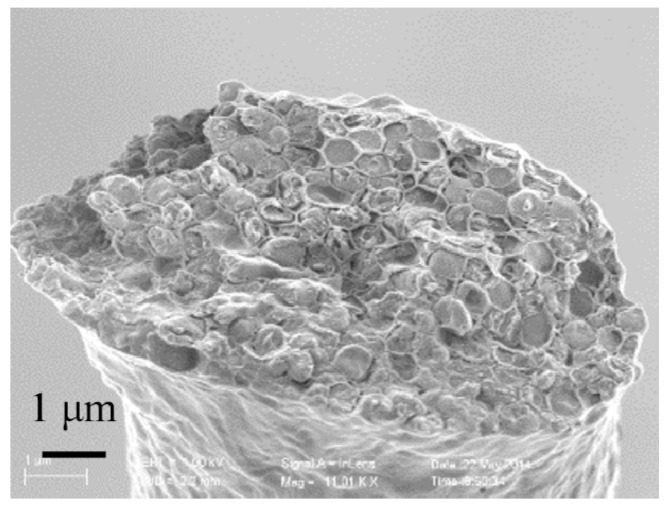
Fracture morphology of a *B. subtilis* thread from the tensile test.

The strength of these threads is less than that of the single filaments because there are many interfaces and inclusions between the different filaments. To investigate the brittle fracture mechanism, the fracture morphology of the thread was observed using the SEM (Figure 17). The cross-section of the thread looks like a honeycomb structure, and the cross-section of each individual filament is hexagonal because the filaments are compressed by shrinkage during drying of the thread. The fracture cross-section indicates that the thread undergoes brittle fracture and does not fracture completely at the positions of the septa in the filaments. Some irregular fracture areas are also observed in the fractured cross-section, which indicates that some defects are present at different positions in the thread. This may be the reason for the low breaking strength of these threads.

## 5. Discussion

To ensure the feasibility and reliability of the system, the noise, vibration, and drift problems must be considered seriously. In the OM, the noise can be divided into two types: ambient noise that comes from the air and from external vibration sources, and system noise that originates from mechanical motion, piezoelectric ceramic motion, creep and nonlinear motion. In the SEM, the main noise sources include: ambient noise from mechanical vibrations; electrical noise from the ac line source (with characteristic frequencies) and the electrostatic charge buildup caused by the strong electromagnetic fields; system noise that originates from the mechanical motion, the piezoelectric ceramic motion, creep and nonlinearity, which are the same sources as those in the OM, where the latter two are the more obvious sources; other sources are derived from vacuum pumping and temperature variations. Additionally, the bidirectional symmetric motor driving system can cause electromagnetic interference.

The presence of noise, vibration and drift may lead to indistinct scanning images and unstable force sensing, necessary and reasonable measures must be taken to eliminate or reduce these adverse effects. The main aspects have been considered as follows: (1) blocking of external vibration sources. The entire system must be installed on a vibration isolation table or the SEM sample stage; (2) strong mechanical connections are required among the vibration isolation table or the SEM sample stage, the experimental system and the other components; (3) the design of the software control system must be optimized by adopting suitable algorithms and through reasonable arrangement of the electronic units, which can effectively weaken some parts of the noise derived from mechanical vibration. The controls for every part are isolated in our system as far as possible to reduce the electromagnetic interference and crosstalk among these parts (e.g., through use of the STM32F103ZET chip as the main embedded controller, which covers all hardware interfaces); (4) ensuring the vacuum compatibility of the materials used for the system components and specimens (where materials with strong magnetism should be avoided); (5) all electronic components should be grounded to prevent any of the conductors from acting as antennae and transmitting noise to the microscope [86]; (6) The use of an appropriate power supply can also effectively reduce the electromagnetic radiation.

After consideration or adoption of these measures, the system drift under different loading conditions improved significantly (see Table 1 and Table 2). When the system was unloaded, the drift speed perpendicular to the tensile direction (*Vy*) was much higher than the drift speed along the tensile direction (*Vx*) because the entire system is suspended and the two specimen stages act like free cantilever beams (Figure 6a). Under the other loading conditions, the *Vx* of the LSS decreases rapidly as a result of the restrictions on the movements of the LSS and the RSS, which are connected by an almost rigid dog bone-shaped specimen (Figure 6c), while the *Vx* and *Vy* values of the RSS are relatively high because of the connection with the force sensor. Longer electron beam irradiation times lead to greater charge accumulation, so the stability of the RSS is not as good as that of the LSS. The results show that the drift speed decreases with time; presumably, this means that the system drift will also remain relatively stable. After approximately 30 min, the average *Vx* is 4.8–15.9 nm/min. This value is also close to the limit of the SEM system that is used for the measurements.

## 6. Conclusions

A novel material testing system that uses hierarchical designs for the in situ mechanical characterization of multiscale materials under OMs and SEMs is established. The m-MTM was proposed to measure the mechanical properties of materials with characteristic lengths ranging from millimeters to several micrometers, and the n-MTM was designed for materials with characteristic lengths ranging from several hundred micrometers to tens of nanometers. Piezoelectric stacks and piezoelectric ceramic tubes were used to realize loading and manipulation with Cartesian coordinates and polar coordinates, respectively. An integrated coarse and fine control module was proposed, and displacements at micrometer and nanometer levels were controlled separately. Therefore, the MTS achieves both multiscale and multifunction features, which is similar to the characteristics of a miniature material mechanical testing machine and a probe-based or MEMS-based testing device. The system capabilities were demonstrated by in situ OM and SEM testing of the system performance and measurement of the material mechanical properties of carbon fibers and metallic wires. In situ multiscale tensile tests of *B. subtilis* filaments and threads revealed the deformation mechanisms and failure behavior at various RH levels, and some proposed methods provided insights to improve their mechanical properties. We expect that this hierarchical design system will be widely used in fundamental research and will also be useful in the study of the multiscale mechanical properties of materials such as biomaterials, composites and self-assembled materials.

## Figures and Tables

**Table 1:** Drift speeds of m-MTM under different loads.

Drift Speed (nm/min)	0		500 μN		11 mN	
	LSS	RSS	LSS	RSS	LSS	RSS
Vx ^1^	<2.5	0	<2.5	0	41.1	11.2
Vy ^2^	57.7	38.9	<−2.5	<−4.9	0	13.2

^1^ *Vx*: drift speed along the tensile direction; ^2^ *Vy*: drift speed perpendicular to the tensile direction.

**Table 2:** Drift speeds of m-MTM under different loading conditions (30 min after initial measurements).

Drift Speed (nm/min)	0		500 μN		11 mN	
	LSS	RSS	LSS	RSS	LSS	RSS
Vx	4.0	−5.6	<2.0	−11.0	<5.3	−26.5
Vy	0	19.5	<3.9	−6.8	<2.7	18.7

**Table 3:** Tensile results of T300 fibers.

Specimen	Tensile Modulus (GPa)	Tensile Strength (GPa)	Elongation (%)
1	218	2.6	1.2
2	203	3.5	1.5
3	208	2.5	1.2
4	204	3.5	1.7

**Table 4:** Tensile results of copper wires.

Specimen	Elastic Modulus (GPa)	Yield Strength (MPa)	Tensile Strength (MPa)	Fracture Strain
1	78.5	225.2	265.4	0.03
2	58.6	202.6	241.5	0.03
3	60.2	230.0	260.1	0.03
4	93.2	240.4	272.2	0.02
